# Enhanced feature dynamic fusion gated UNet for robust retinal vessel segmentation

**DOI:** 10.1038/s41598-025-33694-0

**Published:** 2025-12-26

**Authors:** Yang Yang, Yifeng Li, Jikui Wang, Haibo Zhou, Weihua Zhang, Xing Chen, Tianyun Luan, Wanting Liu, Dashi Ying

**Affiliations:** 1 Changchun University of Science and Technology, Changchun, China; 2 Changchun Sci-Tech University, Changchun, China; 3 Changchun Shikai Technology Industry Co., Ltd, Changchun, China; 4 Shenzhen Customs Industrial Products Testing Technology Center, Shen Zhen, China; 5 The First Bethune Hospital of Jilin University, Changchun, China; 6 Jilin Province FAW General Hospital, Changchun, China; 7 Jilin Agricultural Science and Technology University, Jilin City, China

**Keywords:** Deep learning, Feature dynamic fusion, Retinal vessel segmentation, U-Net, Computational biology and bioinformatics, Engineering, Mathematics and computing

## Abstract

This study proposes a Deep learning model, the Enhanced Feature Dynamic Fusion-Gated U-Net (EFDG-UNet), for retinal vessel segmentation. To address challenges in segmenting small vessels, handling lesion interference, and adapting to multi-scale structures, the model incorporates optimized feature fusion, dynamic selection, and global position modeling. The Feature Navigation Hub (FN-Hub) captures long-range dependencies across multiple encoder layers, improving multi-scale vessel segmentation. The Adaptive Gated Residual Block (AGRB) uses a dynamic gating mechanism to enhance feature selectivity in lesion areas and low-contrast scenarios. The Parallel Focused Attention Module (PFAM) optimizes channel and spatial information for fine-grained vessel features. Experimental validation on DRIVE, CHASE_DB1, and STARE datasets shows that EFDG-UNet achieves state-of-the-art performance, attaining an AUC of 0.9932 and F1-score of 0.8469 on CHASE_DB1, and an AUC of 0.9886 and F1-score of 0.8412 on DRIVE. The model shows improved performance in low-contrast regions and complex vessel structures compared to baseline methods.

## Introduction

Retinal vessel segmentation is crucial in modern medicine. As part of the human microcirculatory system, retinal vessel characteristics help diagnose various ocular and systemic diseases^1^. Structural changes in retinal vessels can indicate early signs of cardiovascular diseases^2^, providing critical evidence for risk assessment of hypertension, coronary artery disease, and stroke. In chronic hypertension, retinal vessels exhibit arterial narrowing and sclerosis, manifesting as “copper-wire” or “silver-wire” appearance. Manual assessment of these subtle changes is subjective, time-consuming, and prone to inter-observer variability, limiting its use in large-scale screening and longitudinal monitoring^3^. Therefore, extracting these clinically valuable features requires precise vessel segmentation. Accurate segmentation enables quantitative analysis of vessel morphology—including caliber, tortuosity, and branching patterns—which is essential for identifying pathological changes and developing personalized treatment plans.

Traditional retinal vessel segmentation requires manual annotation by specialists, which is time-consuming and subjective^4^. Challenges include discontinuities in segmentation, missed detections in low-contrast areas, and difficulties with complex vascular structures. These issues are compounded by imaging device limitations and complex retinal backgrounds.

Deep learning enables automatic feature extraction from annotated datasets, improving segmentation accuracy. Neural networks effectively adapt to complex backgrounds and low-contrast images, capturing multi-scale vascular features without predefined parameters. Models like U-Net^5^ have shown significant advantages in retinal vessel segmentation. Researchers have optimized network architectures through lightweight design, multi-path information fusion, and adaptation to complex vascular structures, enhancing performance with small vessels and low-contrast regions. Challenges remain in segmenting vessels in lesion areas (exudates and hemorrhages)^6^. Large vessel segmentation requires global context, while microvessel segmentation needs finer local features^7^. Current methods often lack specificity in feature extraction strategies, limiting effectiveness in fine-grained tasks.

The key research challenge remains developing models that can flexibly adapt to different vascular types and lesion characteristics while balancing global context with local detail.

The main contributions of this paper are as follows:

- We propose the Adaptive Gated Residual Block (AGRB) with dynamic gating mechanism to improve segmentation performance in lesion areas and low-contrast regions.
- We design the Feature Fusion Hub with Transformer-based attention to fuse multi-scale features and balance global context with local details.
- We introduce the Parallel Focused Attention Module (PFAM) to extract multi-scale vascular features through parallel channel and spatial attention mechanisms.
- We propose the Enhanced Feature Fusion Dynamic Gated U-Net (EFDG-UNet), which integrates AGRB, Feature Fusion Hub, and PFAM to improve segmentation performance in small vessels and lesion-affected areas.

The rest of the paper is organized as follows: Section “Related work” reviews related work; Section “Methods” details the implementation of EFDG U-Net; Section “Experiment” evaluates the proposed method through experiments; and Section “Conclusion” concludes the paper.

## Related work

**Fig. 1:**
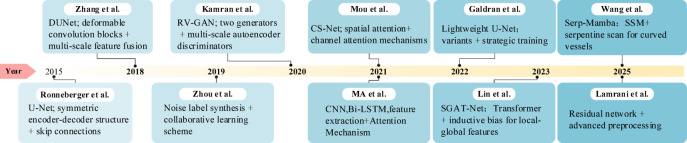
Progression of modeling techniques for retinal vessel Seg-mentation Over the Past Decade.

Recent AI advances have improved automatic retinal vessel segmentation from fundus images, supporting diagnosis and early detection of retinal abnormalities. Figure 1 encapsulates various models developed for retinal vessel segmentation in recent years. In the field of retinal vessel segmentation, the primary challenge initially encountered was the poor performance of traditional machine learning methods in segmenting small and delicate vessels. This limitation arises from the reliance on manually extracted features, which fail to adequately capture the complex morphology of vessels and subtle contrast information. To address this, Ronneberger et al. [^5^] proposed the U-Net model ^1^, the first deep learning architecture specifically designed for biomedical image segmentation. U-Net utilizes a symmetric encoder-decoder structure with skip connections to extract multi-scale features and achieve precise pixel-level localization, which has significantly advanced medical image segmentation.

However, U-Net struggles with vessels in regions of complex morphology and low contrast. Its accuracy in segmenting small vessels is limited, and increasing the network depth may lead to issues such as gradient explosion or vanishing gradients. In response to these limitations, subsequent research has optimized the U-Net architecture. Gegundez-Arias et al. ^8^ enhanced U-Net by incorporating batch normalization, residual modules, and a novel loss function, which improved the separability between pixels and vascular structures. Although these improvements boosted overall performance, the model’s ability to capture small vessels remained limited.

To further address this issue, DUNet ^9^ replaced the standard convolutional layers with deformable convolution blocks, enhancing the encoder’s ability to capture contextual information. Additionally, by merging low- and high-level feature maps, the decoder achieved more accurate localization, significantly improving the segmentation of complex vascular shapes. Mou et al. ^10^ introduced a dual self-attention mechanism, incorporating both spatial and channel attention modules to adaptively integrate local features and global dependencies, thereby improving the model’s sensitivity to small vessels. However, these methods typically introduce additional modules only at the lowest scale, neglecting the capacity to process high-resolution details, resulting in imprecise segmentation of tiny vessels.

To further improve the segmentation of small vessels, Kamran et al. ^11^ proposed RV-GAN, a multi-scale generative architecture featuring two generators and two multi-scale autoencoding discriminators, which more accurately localize and segment tiny vessels. Despite its strong vessel segmentation capabilities, RV-GAN’s relatively low sensitivity suggests that its ability to extract small vessels is still limited. Zhou et al. ^12^ developed a noisy label synthesis procedure and proposed a group learning scheme to improve model performance when trained with imperfect labels. However, these strategies primarily focus on improving label quality and do not dynamically adapt to the characteristics of various vascular morphologies and lesion areas.

Recent advances from 2022 to 2025 have introduced novel perspectives to address these challenges. Galdran et al. ^13^ demonstrated that lightweight U-Net variants with carefully designed training strategies can achieve competitive performance with significantly fewer parameters, challenging the trend toward increasingly complex architectures. The integration of Transformer architectures has marked a paradigm shift, with Lin et al. ^14^ proposing the Stimulus-Guided Adaptive Transformer Network (SGAT-Net) that combines inductive bias with self-attention mechanisms to extract local–global compound features, effectively balancing the extraction of detailed appearance information with contextual understanding.

More recently, state space models (SSMs) have emerged as a promising alternative to Transformers for capturing long-range dependencies in vessel structures. Wang et al. ^15^ introduced Serp-Mamba, which employs a serpentine interwoven adaptive scan mechanism to follow curved vessel structures in ultra-wide-field SLO images, demonstrating competitive performance in maintaining vascular continuity. Most recently, research in 2025 ^16^ has focused on enhancing model convergence and feature learning through residual connections and advanced preprocessing techniques, showing promising results in clinical applications.

Despite these advances, several fundamental challenges persist, particularly in achieving dynamic adaptation to diverse vascular morphologies while maintaining computational efficiency and effectively integrating multi-scale feature fusion with global context modeling.

Although significant progress has been made, several fundamental limitations remain. First, existing methods struggle with fine-grained feature extraction for small and delicate vessels. Second, effective dynamic adaptation mechanisms for diverse vascular morphologies and lesion areas (e.g., exudates and hemorrhages) are lacking. Third, current approaches inadequately balance global contextual information with local detailed features. Addressing these challenges through a model that dynamically adapts to various vascular characteristics while integrating multi-scale feature fusion and global context modeling is crucial for advancing retinal vessel segmentation performance.

## Methods

**Fig. 2:**
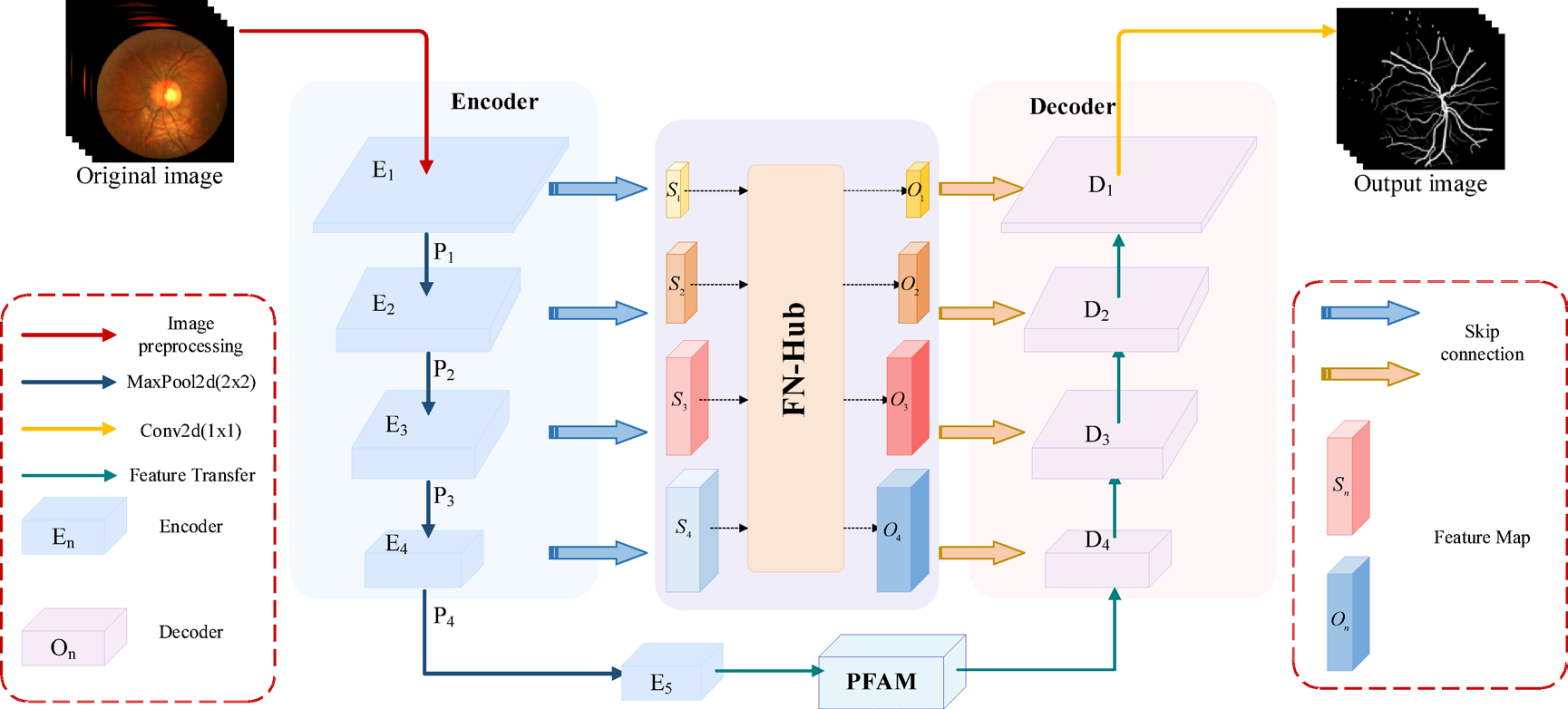
Overview of the EFDG-UNet Architecture.the network is designed for medical image processing, specifically for extracting vascular structures from retinal images. The Encoder compresses the input image into feature representations, the FM-Hub manages and distributes these features, and the Decoder reconstructs the vascular tree from these features.

The proposed EFDG-UNet network, as illustrated in Fig. 2, consists of several key components: a U-shaped structure incorporating downsam-pling and upsampling processes, Adaptive Gated Residual Blocks (AGRB), a Parallel Focused Attention Module (PFAM), and a Feature Fusion Hub (FN-Hub). The network uses ReLU activation and processes preprocessed grayscale images.

The encoder consists of four consecutive AGRBs with downsampling. After each stage, max-pooling halves the spatial dimensions while doubling the channels. Following four downsampling stages, the feature maps are passed to the bottleneck layer. In this layer, the PFAM further processes the features. PFAM leverages an attention mechanism to capture long-range dependencies and location-specific information in the feature maps, enhancing the network’s understanding of global context. Simultaneously, feature maps from each stage of the encoder are passed to the FN-Hub module, which captures global relationships between features and generates enhanced skip connections. The upsampling path of the decoder is symmetric to the encoder structure and consists of four consecutive Up Adaptive Gated Residual Blocks (UpAGRB). During the upsampling process, the spatial dimensions of the feature maps are gradually increased while the number of channels is reduced, restoring the resolution to match the original input. Finally, a 1 × 1 convolutional layer adjusts the number of channels to produce the segmentation results.

### Adaptive gated residual block

**Fig. 3:**
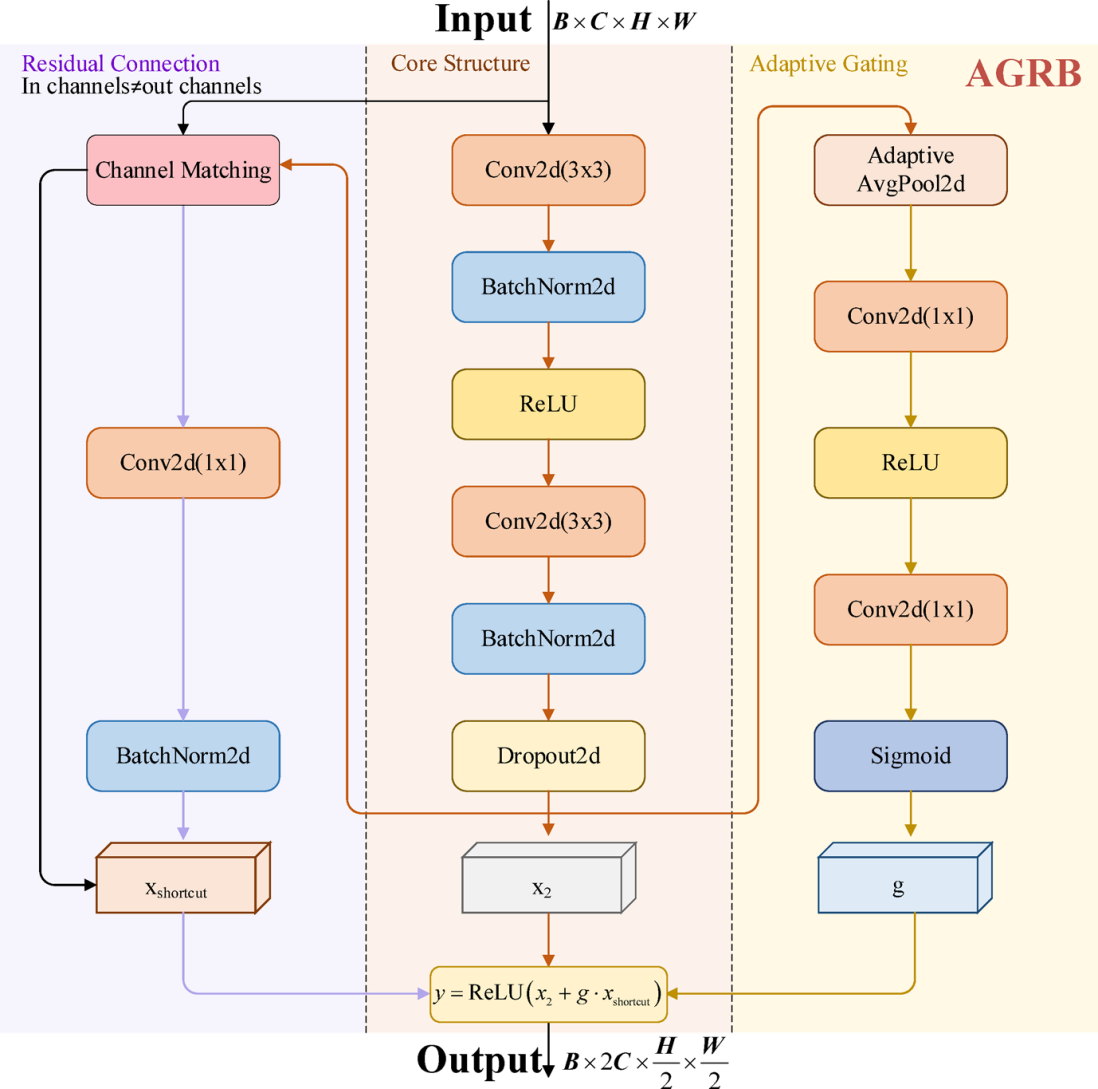
Adaptive gated residual block architecture.

To address the challenges of interference from pathological regions and poor adaptability to low-contrast scenes in retinal vessel segmentation, we propose the AGRB as a replacement for traditional convolutional blocks, As shown in Fig. 3. The AGRB enhances the model’s representational capacity for feature extraction and enhancement through the combination of residual connections and the adaptive gating mechanism. Additionally, Spatial Dropout is introduced to mitigate overfitting. In both the encoder and decoder, AGRBs perform specific tasks: in the encoder, AGRBs progressively extract and compress features, while in the decoder, they are adjusted to restore the image resolution.

In the encoder, the core structure of the AGRB consists of two convolutional layers, each followed by batch normalization and activation functions. First, the input feature map $x$ is processed by the first convolutional layer, followed by batch normalization and a ReLU activation function to produce the intermediate feature map $x_{1}$:

1 $$x_{1} = {\mathrm{ReLU}}\left( {{\mathrm{BN}}_{1} \left( {{\mathrm{Conv}}_{1} \left( x \right)} \right)} \right)$$

Next, the intermediate feature map $x_{1}$ is passed through a second convolutional layer and batch normalization, resulting in the feature map $x_{2}$, as shown in the following equation:

2 $$x_{2} = {\mathrm{BN}}_{2} \left( {{\mathrm{Conv}}_{2} \left( {x_{1} } \right)} \right)$$

A spatial dropout module is applied to $x_{2}$ to reduce noise and enhance regularization. The adaptive gating mechanism of the AGRB extracts global context information via global average pooling, followed by two fully connected layers and a Sigmoid activation function to generate the gating coefficient $g$, as shown in the following equation:

3 $$g = \sigma \left( {{\mathrm{FC}}_{2} \left( {{\mathrm{ReLU}}\left( {{\mathrm{FC}}_{1} \left( {{\mathrm{AdaptiveAvgPool}}\left( {x_{2} } \right)} \right)} \right)} \right)} \right)$$

The gating coefficient $g$ ranges between 0 and 1, adjusting the weight of the residual term. The residual connection performs channel matching on $x$, ensuring its channel number aligns with that of the main branch feature. It is then weighted and added to $x_{2}$, followed by a ReLU activation to obtain the output $y$, the calculation is:

4 $$y = {\mathrm{ReLU}}\left( {x_{2} + g \cdot x_{{{\mathrm{shortcut}}}} } \right)$$

**Fig. 4:**
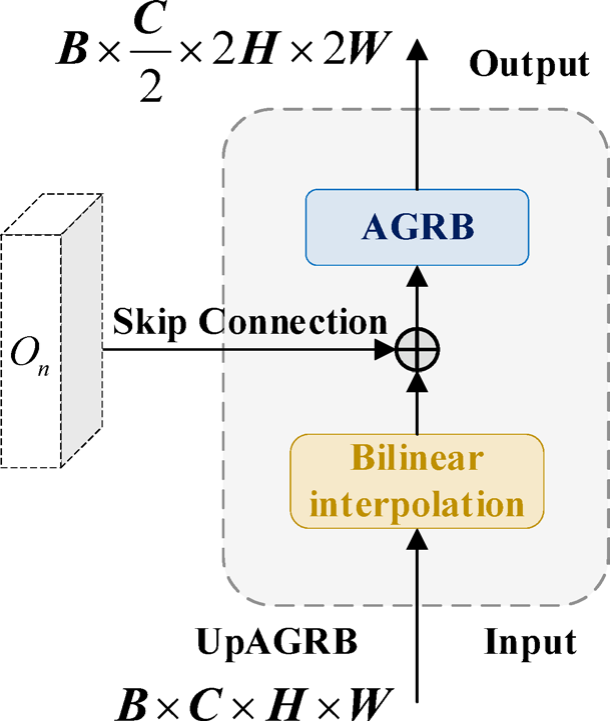
Up adaptive gated residual block architecture.

As shown in Fig. 4, the decoder uses UpAGRB, which adds upsampling and skip connections to the AGRB. The input feature map is first upsampled to half the original image size using bilinear interpolation. Then it is concatenated with the feature map from the corresponding FN-Hub layer to combine semantic information from the encoder. The concatenated features pass through the AGRB module for feature fusion. The adaptive gating mechanism in UpAGRB adjusts the weight of the residual connection based on the input, which helps control how different features contribute during upsampling.

### Parallel feature attention module

**Fig. 5:**
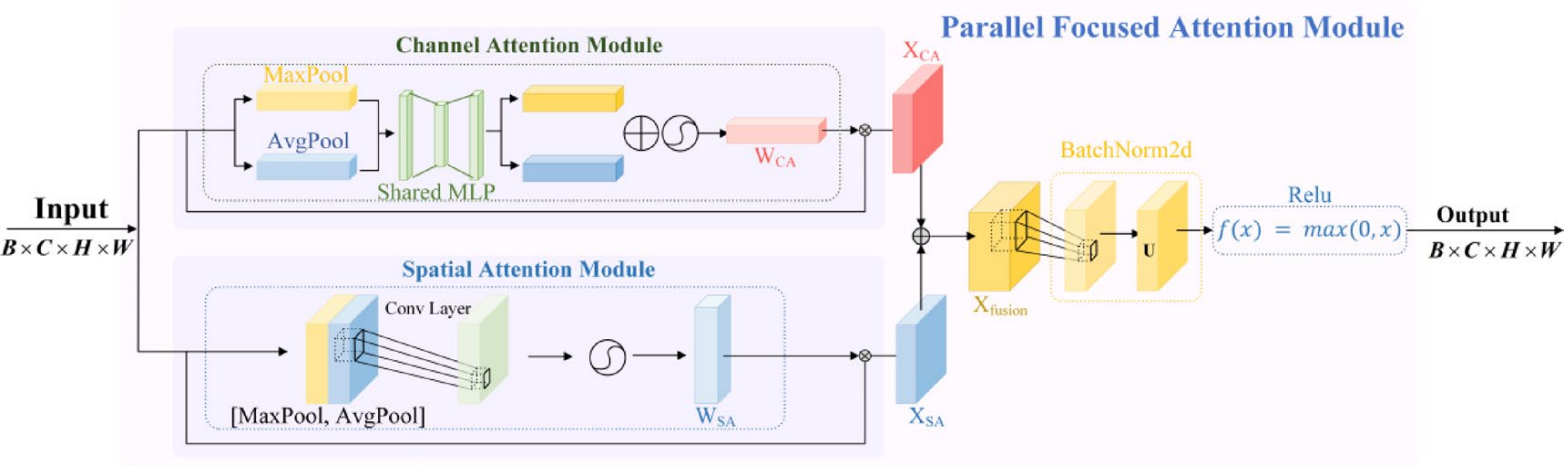
Parallel feature attention module architecture.

PFAM is a module with two parts that work in parallel: a Channel Attention (CA) module and a Spatial Attention (SA) module, as shown in Fig. 5. These two parts process channel information and spatial information at the same time and independently. By handling both types of information separately, PFAM avoids losing important features in either dimension. This parallel processing improves the model’s ability to capture fine-grained features, leading to better segmentation accuracy and robustness.

The Channel Attention Module generates channel importance weights by aggregating the global features of each channel, thereby adjusting the channel-wise feature representation. Specifically, the input feature map $X \in {\mathbb{R}}^{B \times C \times H \times W}$ undergoes global average pooling and max pooling to obtain global descriptors for each channel, denoted as $Y_{{{\mathrm{avg}}}}$ and $Y_{{{\mathrm{max}}}}$, where $Y_{{{\mathrm{avg}}}} ,Y_{{{\mathrm{max}}}} \in {\mathbb{R}}^{B \times C}$. The two descriptors are then summed and passed through a two-layer shared fully connected network for feature compression and expansion, producing the channel weights $W_{{{\mathrm{CA}}}}$, which is given as follows:

5 $${\mathbf{W}}_{{{\mathrm{CA}}}} = \sigma \left( {{\mathbf{W}}_{2} \cdot \delta \left( {{\mathbf{W}}_{1} \cdot {\mathbf{Y}}_{{{\mathrm{avg}}}} } \right)} \right)$$

Here, $W_{1} \in {\mathbb{R}}^{C \times C/r}$ and $W_{2} \in {\mathbb{R}}^{C/r \times C}$ are the weight matrices of the fully connected layers; $r$ denotes the channel compression ratio, $\delta$ represents the ReLU activation function, and $\sigma$ is the Sigmoid activation function. The generated channel weights $W_{{{\mathrm{CA}}}}$ are then multiplied element-wise with the input feature map along the channel dimension, thereby enhancing the channel-wise feature representation, as shown in the following equation:

6 $${\mathbf{X}}_{{{\mathrm{CA}}}} = {\mathbf{X}} \cdot {\mathbf{W}}_{{{\mathrm{CA}}}}$$

The Spatial Attention module captures global spatial information through maximum pooling and average pooling along the spatial dimensions, generating spatial attention weights. The input feature map ${\mathbf{X}}$ undergoes maximum and average pooling along the channel dimension, resulting in two spatial feature maps:

7 $${\mathbf{Y}}_{{{\mathrm{avg}}}} = {\mathrm{AvgPool}}\left( {\mathbf{X}} \right),{\mathbf{Y}}_{{{\mathrm{max}}}} = {\mathrm{MaxPool}}\left( {\mathbf{X}} \right)$$

where $Y_{{{\mathrm{avg}}}} ,Y_{{{\mathrm{max}}}} \in {\mathbb{R}}^{B \times 1 \times H \times W}$. These maps are concatenated along the channel dimension, and the result is passed through a $7 \times 7$ convolutional layer to generate the spatial attention weights, the calculation is:

8 $${\mathbf{W}}_{{{\mathrm{SA}}}} = \sigma \left( {f^{7 \times 7} \left( {\left[ {{\mathbf{Y}}_{{{\mathrm{avg}}}} ;{\mathbf{Y}}_{{{\mathrm{max}}}} } \right]} \right)} \right)$$

where $\left[ ; \right]$ denotes concatenation along the channel dimension, $f^{7 \times 7}$ is a $7 \times 7$ convolution operation, and $\sigma$ is the Sigmoid activation function. Finally, the spatial weights are element-wise multiplied with the input feature map, enhancing the spatial dimension, the calculation is:

9 $${\mathbf{X}}_{{{\mathrm{SA}}}} = {\mathbf{X}} \cdot {\mathbf{W}}_{{{\mathrm{SA}}}}$$

After optimizing both the channel and spatial attention, the module generates the final output feature map through a fusion operation. The results from the channel attention and spatial attention modules are first concatenated along the channel dimension, which is given as follows:

10 $${\mathbf{X}}_{{{\mathrm{fusion}}}} = \left[ {{\mathbf{X}}_{{{\mathrm{CA}}}} ;{\mathbf{X}}_{{{\mathrm{SA}}}} } \right]$$

where $X_{{{\mathrm{fusion}}}} \in {\mathbb{R}}^{B \times 2C \times H \times W}$. The fused feature map undergoes channel compression through a $1 \times 1$ convolutional layer, followed by batch normalization and ReLU activation for further optimization, which is defined as:

11 $${\mathbf{X}}_{{{\mathrm{out}}}} = \delta \left( {{\mathrm{BN}}\left( {f^{1 \times 1} \left( {{\mathbf{X}}_{{{\mathrm{fusion}}}} } \right)} \right)} \right)$$

where $\delta$ represents the ReLU activation function, and “BN” refers to batch normalization. The final feature map $X_{{{\mathrm{out}}}}$ is used for subsequent task processing.

The PFAM module independently optimizes both channel and spatial attention, capturing global channel dependencies and spatial context information in the feature map. The fusion operation further combines the advantages of these two attention mechanisms, providing high-quality feature representations for downstream tasks.

### Feature navigation hub

**Fig. 6:**
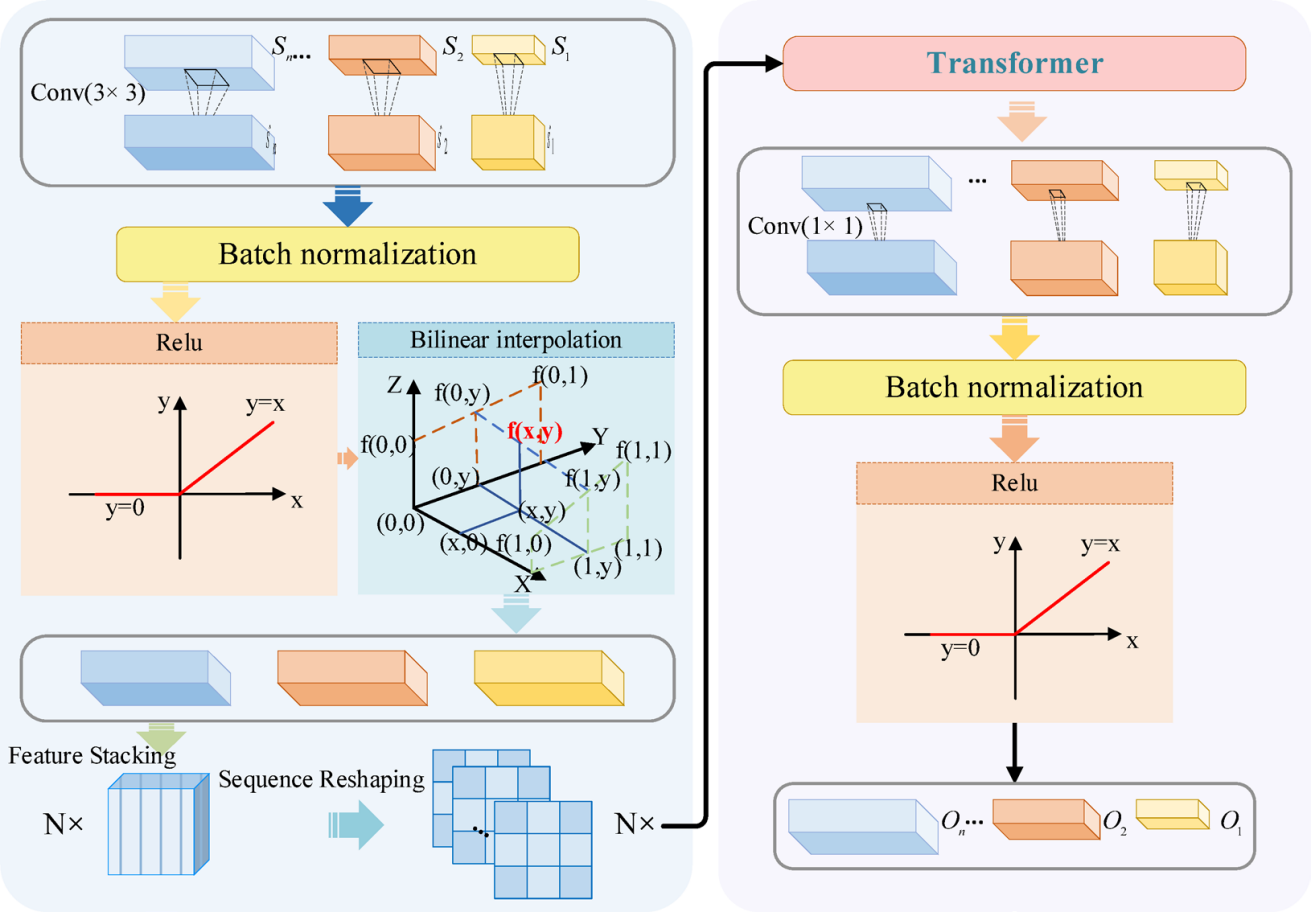
Feature navigation hub architecture.

In this model, we propose a Transformer-based FN-Hub to efficiently integrate feature information from different encoding layers during the decoding phase, as shown in Fig. 6. By incorporating the self-attention mechanism of Transformer, this module captures long-range dependencies between multi-scale features, enhancing its ability to process global context information, especially for fine and coarse vessel structures.

Let the skip connection features from the encoder be denoted as $S_{i}$, where $i = 1,2, \ldots ,n$ represents the number of encoding layers, and $S_{i} \in {\mathbb{R}}^{{C_{i} \times H_{i} \times W_{i} }}$. To unify the channel dimensions, each feature map $S_{i}$ is processed through a 3 × 3 convolution, batch normalization, and ReLU activation, resulting in the standardized feature map $\hat{S}_{i}$, which is defined as:

12 $$\hat{S}_{i} = {\mathrm{ReLU}}\left( {{\mathrm{BN}}\left( {{\mathrm{Conv}}_{3 \times 3} \left( {S_{i} } \right)} \right)} \right)$$

where $\hat{S}_{i} \in {\mathbb{R}}^{{C_{h} \times H_{i} \times W_{i} }}$ is the processed feature, $C_{h}$ is the unified channel count, and Conv, BN, and ReLU represent the convolution, batch normalization, and ReLU activation functions, respectively.

In each decoding layer $D_{j}$, to match the spatial dimensions of the decoder, the feature map $\hat{S}_{i}$ is resized to the target resolution $\left( {H_{j} ,W_{j} } \right)$. This is done via bilinear interpolation, mathematically expressed as:

13 $$\hat{S}_{i}^{j} \left( {x,y} \right) = \mathop \sum \limits_{m = 1}^{2} \mathop \sum \limits_{n = 1}^{2} w_{mn} \cdot \hat{S}_{i} \left( {x_{m} ,y_{n} } \right)$$

where $\hat{S}_{i}^{j} \left( {x,y} \right)$ represents the pixel value at position $\left( {x,y} \right)$ in the target resolution, and $\hat{S}_{i} \left( {x_{m} ,y_{n} } \right)$ refers to the values of the four nearest pixels in the original feature map. The interpolation weight $w_{mn}$ is defined as:

14 $$w_{mn} = \left( {1 - \left| {x - x_{m} } \right|} \right)\left( {1 - \left| {y - y_{n} } \right|} \right)$$

Next, we employ the attention mechanism of the Transformer to capture the dependencies between the multi-scale features $\left\{ {\hat{S}_{i}^{j} } \right\}$. First, the adjusted features are stacked along a new dimension and reshaped into a sequence representation which is given as follows:

15 $$F_{j} = {\mathrm{Stack}}\left( {\left\{ {\hat{S}_{i}^{j} } \right\}} \right) \in {\mathbb{R}}^{{n \times C_{h} \times H_{j} \times W_{j} }}$$

where $F_{j}$ represents the stacked multi-scale features, $n$ is the number of feature layers, and $\left( {H_{j} ,W_{j} } \right)$ is the target resolution.

The features are then reshaped into a tensor with shape $\left( {B,n \times C_{h} ,H_{j} \times W_{j} } \right)$, and the dimensions are rearranged to $\left( {H_{j} \times W_{j} ,B,n \times C_{h} } \right)$ to match the input requirements of the Transformer encoder. After passing through the Transformer encoder, the features integrate global context information, yielding the encoded output, which is given as follows:

16 $$F_{j}^{{{\mathrm{out}}}} = {\mathrm{TransformerEncoder}}\left( {F_{j} } \right)$$

where $F_{j}^{{{\mathrm{out}}}} \in {\mathbb{R}}^{{H_{j} \times W_{j} \times B \times (n \times C_{h} )}}$ contains the multi-scale global context information.

The output features are then restored to the original spatial dimensions $(B,n \times C_{h} ,H_{j} ,W_{j} )$, and an averaging operation is performed across the encoding layers to obtain the fused feature map, the calculation is:

17 $$\hat{F}_{j} = \frac{1}{n}\mathop \sum \limits_{i = 1}^{n} F_{j,i}$$

where $\hat{F}_{j} \in {\mathbb{R}}^{{C_{h} \times H_{j} \times W_{j} }}$ is the fused feature map, and $F_{{\left( {j,i} \right)}}$ denotes the fused result from the *i*-th encoding layer.

The fused feature $\hat{F}_{j}$ is then processed using a 1 × 1 convolution, batch normalization, and ReLU activation to match the required channel count $C_{j}$, resulting in the final output feature map $O_{j}$, which is given as follows:

18 $$O_{j} = {\mathrm{ReLU}}\left( {{\mathrm{BN}}\left( {{\mathrm{Conv}}_{1 \times 1} \left( {\hat{F}_{j} } \right)} \right)} \right)$$

where $O_{j} \in {\mathbb{R}}^{{C_{j} \times H_{j} \times W_{j} }}$ is the generated decoder feature map.

**Fig. 7:**
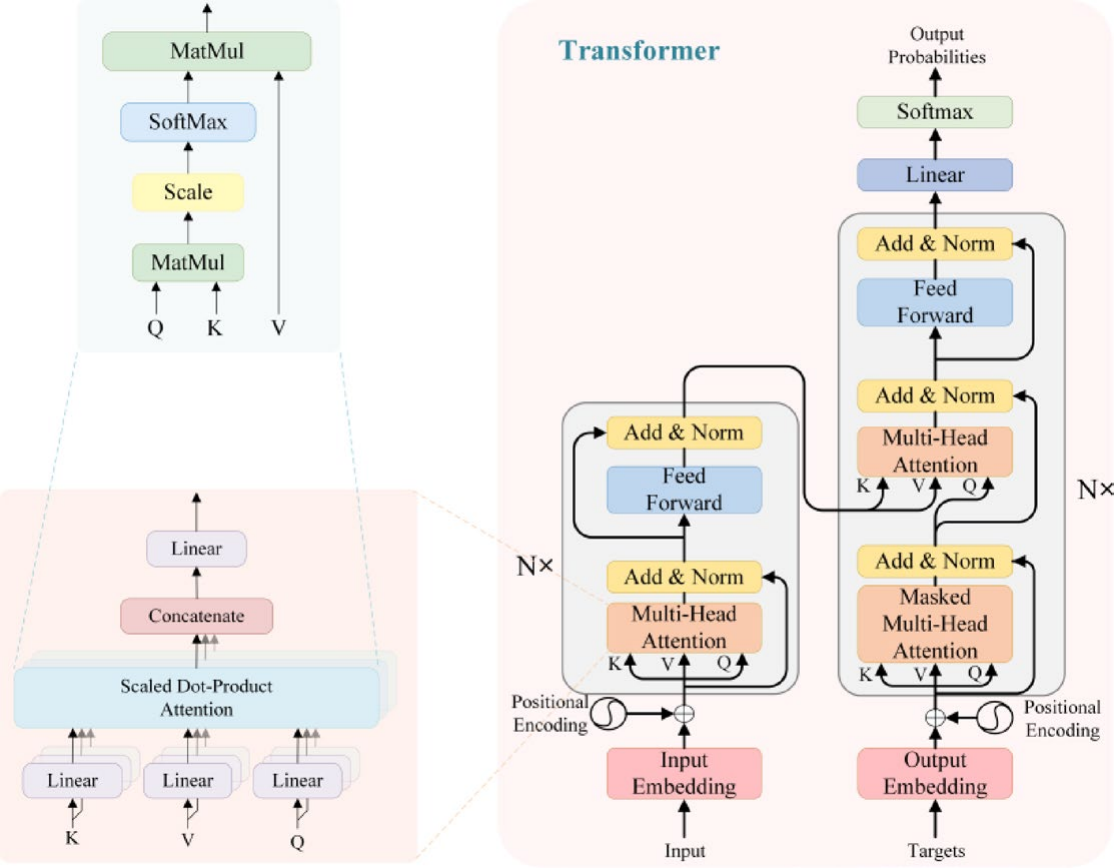
Transformer architecture.

Within the Transformer module, we use the multi-head self-attention mechanism to model the complex dependencies between features. Let the input feature representation be $F$, where $F \in {\mathbb{R}}^{L \times B \times d}$ and $L = H_{j} \times W_{j}$. The core computation for multi-head self-attention is:

19 $${\mathrm{Attention}}\left( {Q,K,V} \right) = {\mathrm{Softmax}}\left( {\frac{{QK^{T} }}{\sqrt d }} \right)V$$

where $Q = FW_{Q}$, $K = FW_{K}$, and $V = FW_{V}$, and $W_{Q} ,W_{K} ,W_{V} \in {\mathbb{R}}^{d \times d}$ are the linear transformation weight matrices for the query, key, and value, respectively. As shown in Fig. 7.

While FN-Hub employs a standard Transformer encoder, its design addresses the data requirements typically associated with Transformer architectures through several key aspects.

First, unlike Vision Transformers that process raw images and must learn visual representations from scratch, FN-Hub operates on pre-extracted encoder features containing rich semantic information. This shifts the learning task from pixel-to-semantic mapping to feature relationship modeling, substantially reducing data requirements.

Second, FN-Hub applies Transformer only at the decoding stage for multi-scale feature integration, rather than replacing the entire architecture. This design inherits the inductive biases (locality and translation invariance) from the pre-trained CNN encoder while maintaining parameter efficiency. The Transformer component builds upon learned representations rather than learning from scratch.

Recent literature validates Transformer-based attention mechanisms on small-scale retinal datasets. Lin et al. ^14^, Lv et al. ^38^, and Chen et al. ^37^ successfully applied similar approaches on datasets of identical scales (DRIVE: 40, CHASE: 28, STARE: 20 images).

## Experiment

### Datasets

**Fig. 8:**
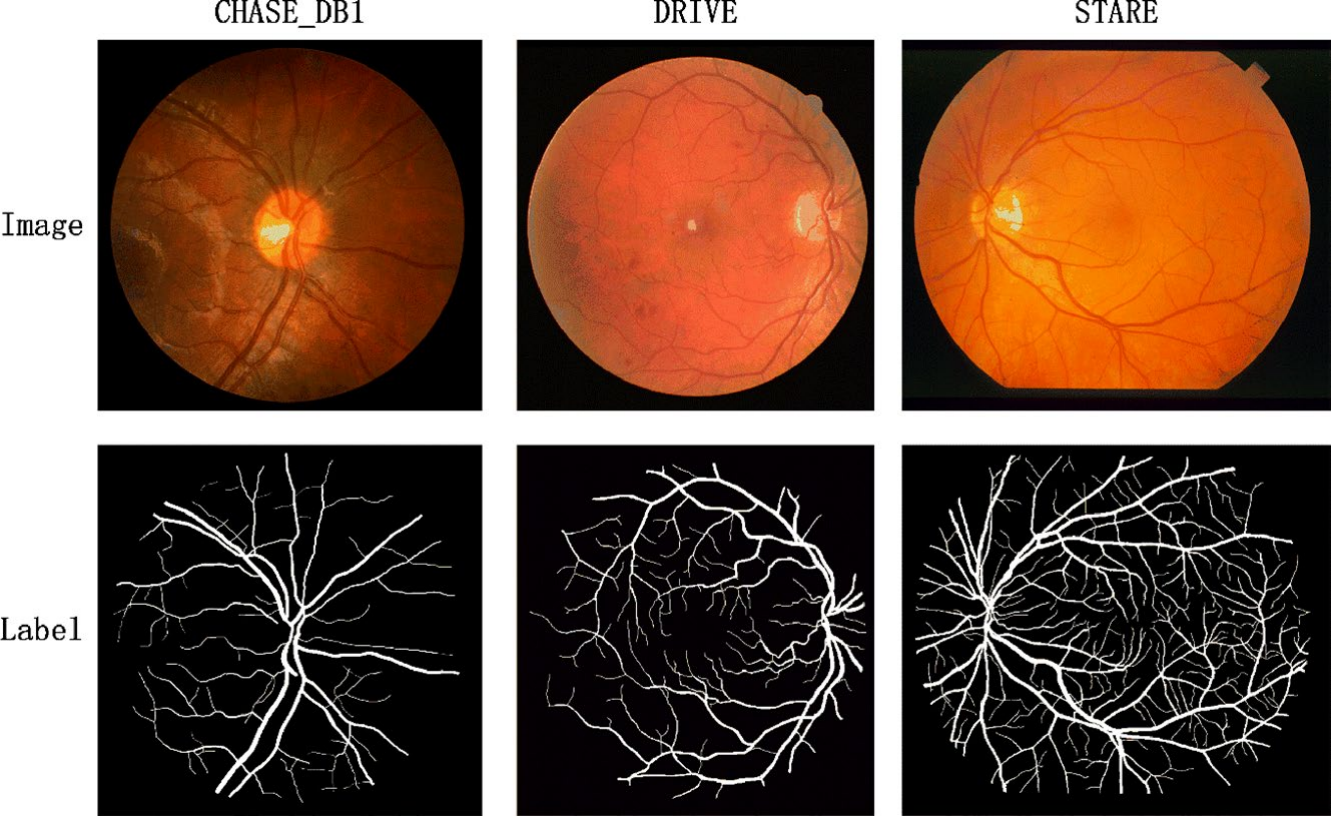
Example samples from DRIVE, CHASE_DB1, and STARE datasets for retinal vessel segmentation.

In our experiments, we utilized the DRIVE, CHASE_DB1, and STARE datasets for training and testing. These three datasets are publicly available and serve as standard benchmarks for retinal vessel segmentation, as shown in Fig. 8.

The DRIVE dataset consists of 40 RGB color fundus images, each with a resolution of 565 × 584 pixels. We divided the dataset into training and validation sets in an 80:20 ratio, using the official training set for the split, while the remaining 20 images were kept as the test set.

The CHASE_DB1 dataset contains 28 RGB color fundus images, with a resolution of 999 × 960 pixels. We assigned the first 14 images to the training set, the following 6 images to the validation set, and the remaining 8 images to the test set.

The STARE dataset comprises 20 RGB color fundus images, each with a resolution of 700 × 605 pixels. We divided the dataset in a 70:20:10 ratio, with the first 10 images used for training, the next 4 for validation, and the remaining 6 for testing.

For all three datasets, we used the annotations from the first expert for evaluating the segmentation performance.

### Image preprocessing

**Fig. 9:**
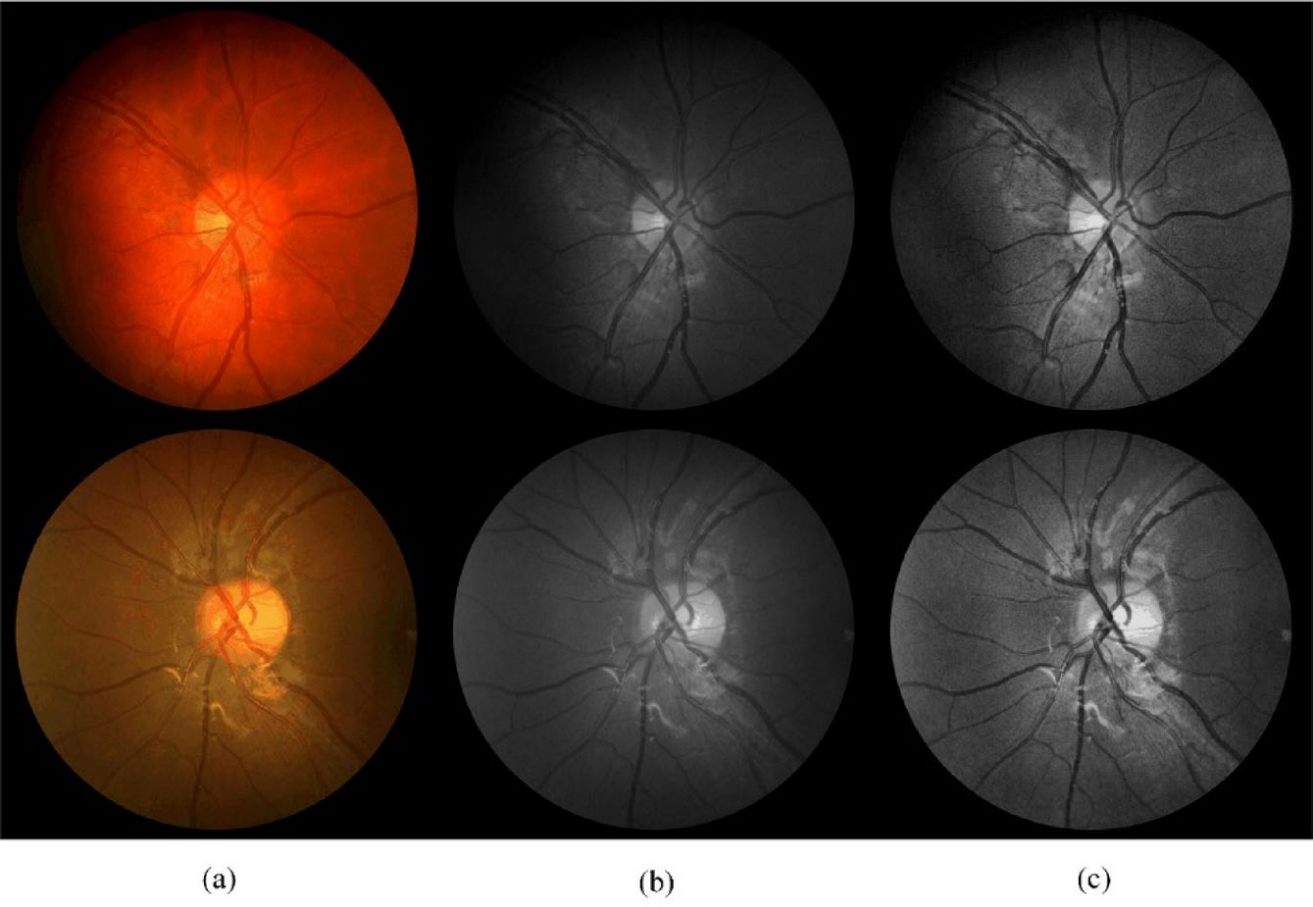
Example images after each preprocessing step. **a** Original Images; **b** Green Channel Extraction; **c** Images After CLAHE Operation.

We apply green channel extraction and CLAHE to enhance retinal image quality. To reduce color variations from different acquisition devices or lighting conditions, we extracted the green channel from RGB images, as it provides superior vessel-background contrast compared to red and blue channels. This single-channel representation also simplifies subsequent processing while preserving essential vessel information.

20 $$I_{{{\mathrm{Gray}}}} = 0.3 \times I_{{{\mathrm{Red}}}} + 0.59 \times I_{{{\mathrm{Green}}}} + 0.11 \times I_{{{\mathrm{Blue}}}}$$

where $I_{{{\mathrm{Gray}}}}$ represents the pixel intensity of the grayscale image, and $I_{{{\mathrm{Red}}}}$, $I_{{{\mathrm{Green}}}}$, and $I_{{{\mathrm{Blue}}}}$ represent the pixel intensities of the red, green, and blue channels, respectively. To further enhance vessel-background contrast, we applied CLAHE preprocessing. CLAHE adaptively equalizes local contrast by redistributing intensity values within non-overlapping image regions, with a clip limit to suppress noise amplification. We set the clip limit to 2.0 and divided images into 8 × 8 grids for region-wise processing. As shown in Fig. 9, these preprocessing steps significantly improved vessel visibility, with small vessels becoming particularly distinguishable from the background.

To enhance the model’s generalization ability, we applied various data augmentation techniques to the training set. Geometric transformations included horizontal and vertical flips, rotations at 15° and 170°, elastic deformation with large amplitude, and optical distortion with limited distortion strength. Photometric transformations included Gaussian blurring with kernel sizes varying between 3 and 7, Gaussian noise with intensity ranging from 10 to 50, and brightness/contrast adjustment within ± 20%. All augmented images were then cropped into 48 × 48 non-overlapping patches for training.

### Image segmentation performance evaluation metrics

In image segmentation tasks, the following performance metrics were used to provide a comprehensive evaluation of segmentation effectiveness: Intersection over Union (IoU), Accuracy (ACC), Sensitivity (Se), Specificity (Sp), F1 Score (F1), and Area Under the Curve (AUC). The specific definitions are as follows:

21 $${\mathrm{ACC}} = \frac{{{\mathrm{TP}} + {\mathrm{TN}}}}{{{\mathrm{TP}} + {\mathrm{FP}} + {\mathrm{TN}} + {\mathrm{FN}}}}$$

22 $${\mathrm{Se}} = \frac{{{\mathrm{TP}}}}{{{\mathrm{TP}} + {\mathrm{FN}}}}$$

23 $${\mathrm{Sp}} = \frac{{{\mathrm{TN}}}}{{{\mathrm{TN}} + {\mathrm{FP}}}}$$

24 $$F1 = \frac{2TP}{{2TP + FP + FN}}$$

We used five standard metrics to evaluate model performance: Accuracy (ACC), Sensitivity (Se), Specificity (Sp), F1 Score, and Area Under the Curve (AUC). ACC represents the proportion of correctly classified pixels. Se measures the ability to identify vessel pixels (true positive rate), while Sp measures the ability to correctly identify background pixels (true negative rate). F1 Score balances precision and sensitivity, and AUC evaluates overall performance across different decision thresholds.

### Experimental details

We evaluated model performance on three public datasets (CHASE, DRIVE, and STARE), comparing results with state-of-the-art methods. Ablation experiments were conducted to analyze each module’s contribution. The model was implemented in PyTorch and trained using the Adam optimizer with an initial learning rate of 0.0001. Detailed experimental environment and hyperparameter settings are listed in Table 1. We employed the ReduceLROnPlateau strategy to dynamically adjust the learning rate, reducing it to 10% when validation loss showed no improvement. Training was performed for 100 epochs with a batch size of 16 and cross-entropy loss function.

**Table 1:** Experimental environment and hyperparameter settings.

Content	Details
Test datasets	CHASE, DRIVE, STARE
Implementation platform	Python 3.10
Optimizer	Adam
Initial learning rate	0.0001
Batch size	16
Loss function	Binary cross-entropy loss
Total epochs	100 epochs

### Comparisons with state of the art models

Table 2 presents a performance comparison of various algorithms on the CHASE_DB1 dataset, including classical methods such as U-Net, AttentionU-Net, LadderNet, and ResUNet++, as well as recent advanced models. As shown, EFDG-UNet outperforms the other models on the CHASE_DB1 dataset, achieving superior Acc, Sp, AUC, and F1 score.

**Table 2:** Comparison of different algorithms on the CHASE_DB1 dataset.

Methods	Date	ACC	SE	SP	AUC	F1
U-Net^1^	2015	0.9684	0.7612	0.9885	0.9812	0.8075
Attention U-Net^17^	2018	0.9753	0.8035	0.9887	0.9896	0.8248
LadderNet^18^	2019	0.9656	0.7978	0.9818	0.9839	0.8031
ResUNet++^19^	2019	0.9732	0.7425	**0.9924**	0.9884	0.8095
HRNet^20^	2019	0.9752	0.8151	0.9885	0.9908	0.8346
AG-NET^21^	2019	0.9743	0.8186	0.9848	0.9863	–
UNet3+ ^22^	2020	0.9746	0.8121	0.9881	0.9905	0.8309
RVSeg-Net^23^	2020	0.9726	0.8069	0.9836	0.9833	–
BEFD^24^	2021	0.9755	0.8573	0.9835	0.9905	–
SA-UNet^25^	2020	0.9755	0.8573	0.9835	0.9905	–
VSSC-Net^26^	2021	0.9633	0.7233	0.9865	0.9706	–
SCS-Net^27^	2021	0.9744	0.8365	0.9839	0.9867	–
MMDC-Net^28^	2022	0.9646	0.8440	0.9728	0.9541	–
D-MNet^31^	2022	0.9714	0.8541	0.9794	0.9677	0.7900
MBSNet^32^	2023	0.9759	0.8162	0.9892	0.9892	0.8386
BCU-Net^35^	2023	0.9731	0.8463	0.9817	0.9814	0.7991
SDDC-Net^34^	2023	0.9727	**0.8627**	0.9808	0.9894	0.8047
CFFANet^37^	2024	0.9590	–	0.9840	–	0.8420
TCDDU-Net^38^	2024	0.9723	0.8187	0.9823	0.9850	0.7838
DMSU-Net++ ^39^	2025	–	0.8281	0.9880	0.9836	0.8281
MVM-UNet^40^	2025	**0.9762**	0.8305	0.9860	–	–
EFDG-UNet	2025	**0.9788**	0.8405	0.9893	**0.9932**	**0.8469**

EFDG-UNet achieves accuracy of 0.9788, specificity of 0.9893, AUC of 0.9932, and F1 score of 0.8469, leading in overall performance. Compared to the latest 2024–2025 models, EFDG-UNet demonstrates superior accuracy over MVM-UNet with an accuracy of 0.9762 and TCDDU-Net with an accuracy of 0.9723. The sensitivity of 0.8405 is slightly lower than the sensitivity of SDDC-Net of 0.8627 and D-MNet of 0.8541, but higher than MVM-UNet with a sensitivity of 0.8305, DMSU-Net++ with a sensitivity of 0.8281, and TCDDU-Net with a sensitivity of 0.8187, and it shows a 10.5% and 9.8% improvement over U-Net and ResUNet++ in terms of sensitivity, respectively, indicating significant progress in detecting small vessels and complex details. Regarding specificity, EFDG-UNet leads most comparison models with a specificity of 0.9893, surpassing DMSU-Net++ with a specificity of 0.9880, MVM-UNet with a specificity of 0.9860, and TCDDU-Net with a specificity of 0.9823, second only to ResUNet++ with a specificity of 0.9924 and MBSNet with a specificity of 0.9892. This suggests its high reliability in distinguishing vessels from background regions, effectively reducing false positives and enhancing segmentation accuracy. With an AUC of 0.9932, EFDG-UNet outperforms TCDDU-Net with an AUC of 0.9850 and DMSU-Net++ with an AUC of 0.9836. The F1 score of 0.8469 is the highest among all models, exceeding CFFANet with an F1 score of 0.8420, DMSU-Net++ with an F1 score of 0.8281, TCDDU-Net with an F1 score of 0.7838, as well as MBSNet with an F1 score of 0.8386, and is also higher than Attention U-Net with an F1 score of 0.8248 and HRNet with an F1 score of 0.8346, demonstrating a good balance between precision and recall and reflecting its superior overall performance.

Table 3 summarizes the performance of EFDG-UNet and other leading algorithms on the DRIVE dataset. The comparison results show that EFDG-UNet achieves a favorable balance across several key metrics, showcasing its potential as an advanced segmentation model.

**Table 3:** Comparison of different algorithms on the DRIVE dataset.

Methods	Date	ACC	SE	SP	AUC	F1
U-Net^1^	2015	0.9691	0.7948	**0.9860**	0.9845	0.8172
Attention U-Net^17^	2018	0.9661	0.8092	0.9813	0.9776	0.8015
LadderNet^18^	2019	0.9561	0.7856	0.9810	0.9793	0.8202
ResUNet++ ^19^	2019	0.9662	0.7856	0.9837	0.9801	0.8015
HRNet^20^	2019	0.9683	0.8152	0.9831	0.9855	0.8171
AG-NET^21^	2019	0.9692	0.8100	0.9848	0.9856	–
UNet3+ ^22^	2020	0.9702	0.8081	**0.9860**	0.9860	0.8254
RVSeg-Net^23^	2020	0.9681	0.8107	0.9845	0.9817	0.8267
BEFD^24^	2021	0.9701	0.8215	0.9845	0.9867	–
SA-UNet^25^	2020	0.9698	0.8212	0.9840	0.9864	–
VSSC-Net^26^	2021	0.9627	0.7827	0.9821	0.9789	–
SCS-Net^27^	2021	0.9697	0.8289	0.9838	0.9837	–
MMDC-Net^28^	2022	0.9607	0.8074	0.9755	0.9613	–
UCR-Net^29^	2022	0.9671	0.8120	0.9822	0.9847	–
DCA-CNN^30^	2022	0.9630	**0.8745**	0.9823	0.9670	–
D-MNet^31^	2022	0.9683	0.8363	0.9811	–	0.8211
MBSNet^32^	2023	0.9692	0.8234	0.9834	0.9873	0.8234
DCSAU-Net^33^	2023	0.9667	0.8376	0.9792	0.9827	0.8139
SDDC-Net^34^	2023	0.9704	0.8603	0.9808	0.9706	0.8289
BCU-Net^35^	2023	0.9667	0.8142	0.9816	0.9791	0.8096
GDF-Net^36^	2023	0.9622	0.8291	0.9852	0.9859	0.8302
CFFANet^37^	2024	0.9560	–	0.9760	–	0.8290
TCDDU-Net^38^	2024	0.9698	0.8258	0.9838	0.9868	0.8265
DMSU-Net + + ^39^	2025	–	0.8374	0.9845	0.9786	0.8275
MVM-UNet^40^	2025	0.9683	0.8547	0.9786	–	–
EFDG-UNet	2025	**0.9736**	0.8438	0.9856	**0.9886**	**0.8412**

From the perspective of accuracy, EFDG-UNet achieves a value of 0.9736, the highest among all compared models, surpassing TCDDU-Net with an accuracy of 0.9698, MVM-UNet with an accuracy of 0.9683, comparable to high-performance models such as UNet3+ and SDDC-Net, and significantly outperforming traditional models with an accuracy of 0.9691 for U-Net and 0.9561 for LadderNet. This indicates that EFDG-UNet offers higher precision in overall segmentation tasks. In terms of specificity, EFDG-UNet scores 0.9856, leading the field and surpassing recent models including DMSU-Net++ with a specificity of 0.9845, TCDDU-Net with a specificity of 0.9838, and MVM-UNet with a specificity of 0.9786, and only slightly behind a few models such as ResUNet++ with a specificity of 0.9837, which reflects its efficiency in recognizing background regions. Regarding sensitivity, EFDG-UNet performs at 0.8438, higher than DMSU-Net++ with a sensitivity of 0.8374 and TCDDU-Net with a sensitivity of 0.8258, but slightly lower than MVM-UNet with a sensitivity of 0.8547, substantially outperforming classical methods such as U-Net with a sensitivity of 0.7948 and some modern models such as ResUNet++ with a sensitivity of 0.7856, demonstrating its greater capability in detecting small vessels. Although its sensitivity is slightly lower than the sensitivity of SDDC-Net of 0.8603 and DCA-CNN of 0.8745, EFDG-UNet’s strong balance in specificity and accuracy makes it more balanced in overall performance.

EFDG-UNet achieves an AUC of 0.9886 and an F1 score of 0.8412, both ranking among the top performers. The AUC of 0.9886 is the highest among all models, outperforming TCDDU-Net with an AUC of 0.9868, DMSU-Net++ with an AUC of 0.9786, and MBSNet with an AUC of 0.9873. The F1 score of 0.8412 is also the highest, exceeding DMSU-Net++ with an F1 score of 0.8275, TCDDU-Net with an F1 score of 0.8265, and CFFANet with an F1 score of 0.8290. The AUC reflects the model’s robustness across different thresholds, while the F1 score highlights the harmony between precision and recall. Compared to other recent models, EFDG-UNet demonstrates superior segmentation performance, particularly in terms of overall capability.

EFDG-UNet excels in multiple key metrics, especially in Acc and comprehensive segmentation performance. Its high Se and Sp values indicate that the model effectively balances segmentation of both small vessels and background regions. Its outstanding AUC and F1 scores further validate its robustness and reliability, making it one of the leading methods for retinal vessel segmentation.

As shown in Table 4, EFDG-UNet achieves an accuracy of 0.9637, specificity of 0.9769, an AUC of 0.9842, and an F1 score of 0.8376, particularly excelling in specificity and AUC. However, compared to other methods, EFDG-UNet’s overall performance still has room for improvement, especially in terms of sensitivity. Although its sensitivity is 0.8575, which is satisfactory, it surpasses MVM-UNet with a sensitivity of 0.8178 and TCDDU-Net with a sensitivity of 0.7920, it remains slightly lower than that of several other methods.

**Table 4:** Comparison of different algorithms on the STARE dataset.

Methods	Date	ACC	SE	SP	AUC	F1
U-Net^1^	2015	0.9738	0.7814	0.9897	0.9892	0.8138
Attention U-Net^17^	2018	0.9719	0.8009	0.9859	0.9886	0.8084
ResUNet++ ^19^	2019	0.968	0.8173	0.9803	0.9854	0.7906
HRNet^20^	2019	0.9731	0.8159	0.986	0.9885	0.8159
UNet3+ ^22^	2020	0.9731	0.8064	0.9869	0.9889	0.8153
SCS-Net^27^	2021	0.9736	0.8207	0.9839	0.9877	–
MMDC-Net^28^	2022	0.9591	0.8509	0.9689	0.9687	–
D-MNet^31^	2022	0.9732	0.8272	0.9847	–	0.8196
MBSNet^32^	2023	0.9739	0.8125	0.9871	**0.9898**	0.8213
DCSAU-Net^33^	2023	0.9726	0.819	0.9852	0.9872	0.8162
SDDC-Net^34^	2023	0.9669	0.8268	0.9789	0.9845	0.7965
BCU-Net^35^	2023	0.9701	0.8500	0.9807	0.9807	0.8223
GDF-Net^36^	2023	0.9653	0.7616	**0.9957**	0.9889	0.8022
CFFANet^37^	2024	0.9630	–	0.9800	–	**0.8400**
TCDDU-Net^38^	2024	**0.9740**	0.7920	0.9884	0.9856	0.8163
MVM-UNet^40^	2025	0.9715	0.8178	0.9839	–	–
EFDG-UNet	2025	0.9637	**0.8575**	0.9769	0.9842	0.8376

The sensitivity of ResUNet++ and SCS-Net is 0.8173 and 0.8207, respectively, slightly higher than EFDG-UNet’s, suggesting that these methods may be better suited for precise target detection in certain tasks. MMDC-Net, with a sensitivity of 0.8509, is close to EFDG-UNet, indicating comparable performance in target detection. BCU-Net achieves a sensitivity of 0.8500, also comparable to EFDG-UNet.

In terms of specificity, GDF-Net stands out with a specificity of 0.9957, significantly outperforming other methods. This suggests that GDF-Net is particularly effective at reducing false positives. However, GDF-Net’s accuracy and F1 score are 0.9653 and 0.8022, respectively, with the lower F1 score indicating an imbalance in its overall performance despite its high specificity. EFDG-UNet’s specificity of 0.9769 is lower than TCDDU-Net with a specificity of 0.9884 and MBSNet with a specificity of 0.9871, but higher than MVM-UNet with a specificity of 0.9839 and CFFANet with a specificity of 0.9800. In contrast, EFDG-UNet maintains a more balanced performance across accuracy, specificity, and F1 score, without overemphasizing any single metric, demonstrating more stable and comprehensive performance.

U-Net and Attention U-Net achieve accuracies of 0.9738 and 0.9719, respectively, along with TCDDU-Net with an accuracy of 0.9740 and MBSNet with an accuracy of 0.9739, slightly higher than EFDG-UNet. However, their performance in sensitivity and F1 score is somewhat weaker, particularly Attention U-Net, which has an F1 score of 0.8084. Despite the higher accuracy, their overall performance balance is slightly inferior. Notably, EFDG-UNet’s F1 score of 0.8376 is competitive, slightly lower than CFFANet with an F1 score of 0.8400, but exceeding BCU-Net with an F1 score of 0.8223, MBSNet with an F1 score of 0.8213, and TCDDU-Net with an F1 score of 0.8163. Therefore, while other models may outperform EFDG-UNet in specific metrics, EFDG-UNet demonstrates a more balanced performance across multiple key indicators.

In terms of AUC, EFDG-UNet achieves 0.9842, which is competitive with top-performing models, though slightly lower than MBSNet with an AUC of 0.9898 and TCDDU-Net with an AUC of 0.9856. In summary, EFDG-UNet demonstrates exceptional overall performance in accuracy, AUC, and specificity, particularly excelling in specificity and AUC. Although its sensitivity is slightly lower, EFDG-UNet remains highly competitive, especially for medical image segmentation tasks that require balanced and robust overall performance. In comparison, while other methods may excel in specific metrics, their overall performance tends to favor certain aspects, lacking the comprehensive balance that EFDG-UNet offers.

### Vascular analysis

**Fig. 10:**
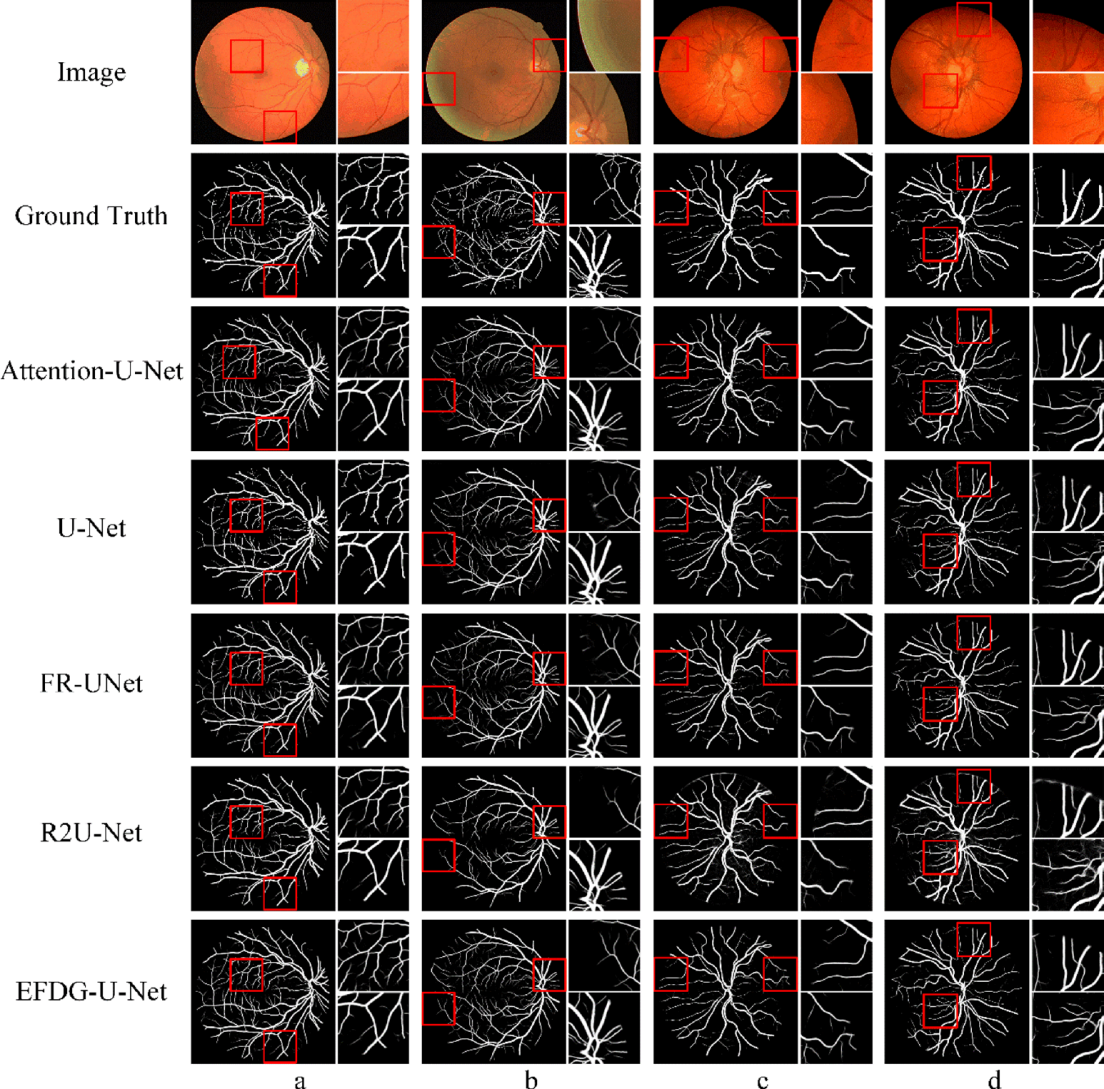
Comparison of the segmentation results of EFDG-UNet and other methods on the CHASE_DB1 and DRIVE datasets.

To further demonstrate the segmentation performance of EFDG-UNet on complex small vessel shapes, we conducted comparative experiments on the CHASE_DB1 and STARE datasets, which contain numerous small vessels with intricate shapes. Figure 10 shows the original images and manual annotations from different datasets, along with the segmentation results from Oktay et al. ^17^, Ronneberger et al. ^1^, Liu et al. ^41^, Alom et al. ^42^, and our proposed EFDG-UNet. Specifically, (a) and (b) are examples from the DRIVE dataset, and (c) and (d) are from the CHASE_DB1 dataset. EFDG-UNet demonstrates significant advantages in segmenting complex vascular regions. For larger vessels, EFDG-UNet exhibits greater coherence and completeness compared to models such as Attention-U-Net, R2U-Net, and FR-UNet, accurately preserving the overall vessel morphology. In the segmentation of smaller vessels, EFDG-UNet not only accurately identifies vessel branches and junctions but also significantly reduces misclassifications, avoiding false-positive segmentation of background regions as vessels. Overall, EFDG-UNet exhibits stronger robustness and higher segmentation precision in fine-grained vascular segmentation tasks.

**Fig. 11:**
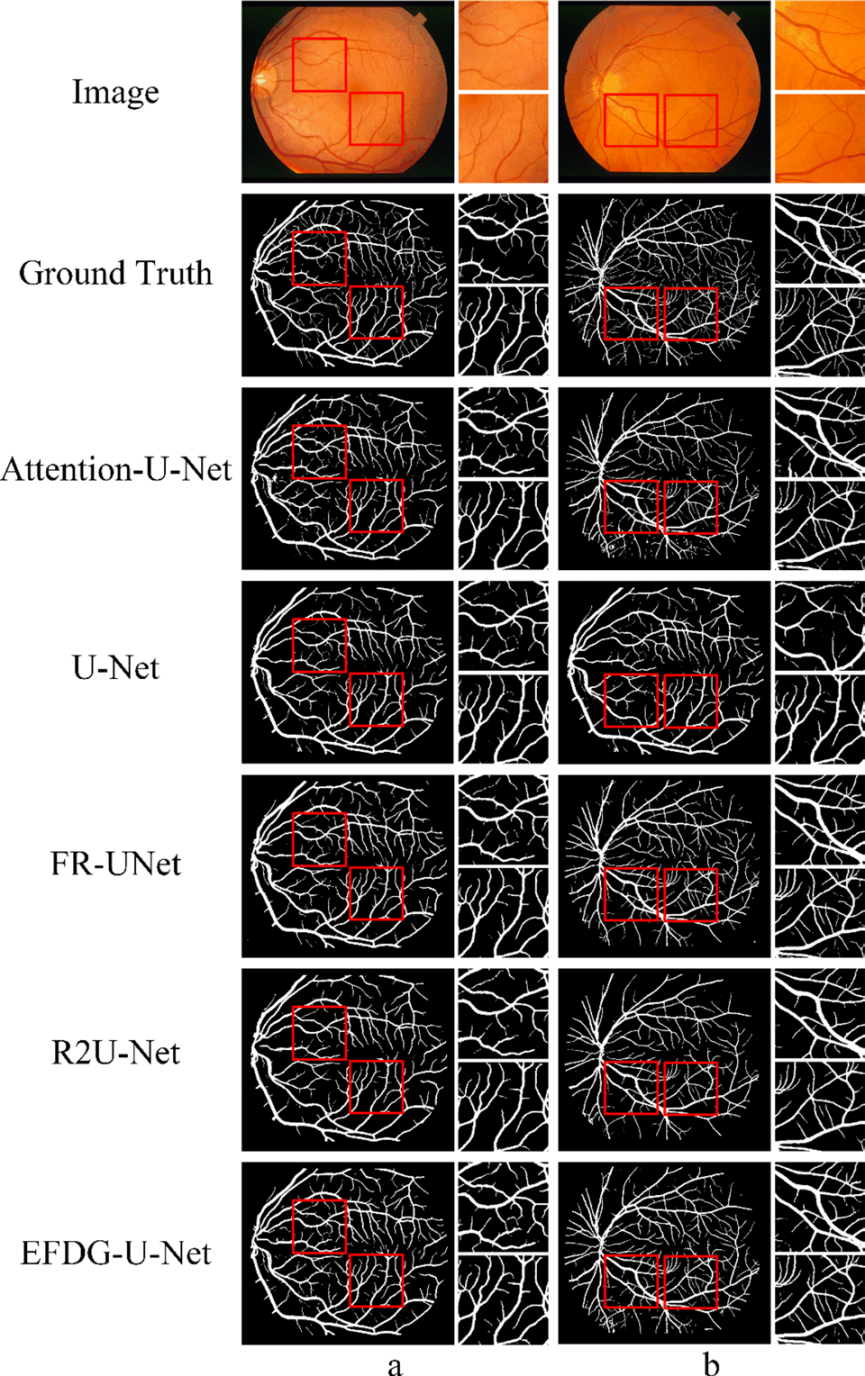
Comparison of the binarized segmentation results of EFDG-UNet and other methods on the STARE dataset.

To better illustrate the segmentation performance, we present binarized segmentation results on the STARE dataset. Figure 11 shows the segmentation results of all models, including a comparison between EFDG-UNet and other methods.

As shown in Fig. 11, EFDG-UNet continues to demonstrate clear advantages in vascular extraction tasks, particularly in segmenting small vessels and maintaining vessel connectivity. Compared to other methods, EFDG-UNet more accurately preserves the continuity and integrity of vessels when processing complex vascular networks. Specifically, in the segmentation of small vessels, EFDG-UNet clearly distinguishes vessels from background regions, reducing false positives and missed detections while preventing the background from being incorrectly segmented as vessels. Additionally, EFDG-UNet shows higher coherence in extracting larger vessels, preserving both the vessel’s diameter variations and structural features more effectively than other models such as U-Net and ResUNet.

### Ablation study

An ablation study was conducted to assess the individual components of EFDG-UNet on the CHASE_DB1, DRIVE, and STARE datasets, with the original U-Net serving as the baseline model.

As shown in Table 5, the ablation experiments on the CHASE_DB1 dataset demonstrate the contribution of each module to the overall performance of EFDG-UNet.

**Table 5:** Ablation experiment for EFDG-UNet on CHASE_DB1 dataset.

Argument	Acc	Se	Sp	AUC	F1
Unet	0.9734	0.8791	0.9806	0.9898	0.8218
AGRB	0.9773	0.8287	0.9886	0.9919	0.8362
PFAM + AGRB	0.9773	0.8181	0.9894	0.9920	0.8342
FN-Hub + AGRB	0.9776	0.8540	0.9870	0.9925	0.8418
EFDG-UNet	0.9788	0.8405	0.9893	0.9932	0.8469

The baseline U-Net model achieved an accuracy of 0.9734, sensitivity of 0.8791, specificity of 0.9806, AUC of 0.9898, and F1 score of 0.8218. After introducing the AGRB module, accuracy improved to 0.9773, specificity increased to 0.9886, and F1 score rose to 0.8362. However, sensitivity decreased to 0.8287. When combining PFAM with AGRB, specificity further increased to 0.9894 and AUC reached 0.9920, but sensitivity continued to decline to 0.8181. The combination of FN-Hub and AGRB achieved an accuracy of 0.9776, with sensitivity improving to 0.8540 and F1 score reaching 0.8418. The complete EFDG-UNet model, integrating all three modules, achieved the best overall performance with an accuracy of 0.9788, sensitivity of 0.8405, specificity of 0.9893, AUC of 0.9932, and F1 score of 0.8469. It is noteworthy that EFDG-UNet exhibits a decrease in sensitivity (0.8405 vs. 0.8791) compared to the baseline U-Net, which reflects the classical sensitivity–specificity trade-off in medical image segmentation. The model achieves substantially higher specificity (0.9893 vs. 0.9806) at the cost of slightly lower sensitivity. Importantly, the improvements in F1 score (0.8469 vs. 0.8218) and AUC (0.9932 vs. 0.9898) demonstrate that EFDG-UNet achieves superior overall performance and better balance between precision and recall. This trade-off is particularly evident in the ablation results: while AGRB initially reduces sensitivity to 0.8287, the FN-Hub module effectively mitigates this issue by improving sensitivity to 0.8540 in the FN-Hub + AGRB configuration.

As shown in Table 6, the ablation experiments on the DRIVE dataset further validate the effectiveness of each module in EFDG-UNet.

**Table 6:** Ablation experiment for EFDG-UNet on DRIVE dataset.

Argument	Acc	Se	Sp	Auc	F1
UNet	0.9691	0.7948	0.9860	0.9845	0.8172
AGRB	0.9716	0.8151	0.9867	0.9875	0.8297
FN_Hub + AGRB	0.9730	0.8561	0.9839	0.9887	0.8402
PFAM + AGRB	0.9725	0.8584	0.9831	0.9889	0.8380
EFDG-UNet	0.9736	0.8438	0.9856	0.9886	0.8412

The baseline U-Net model achieved an accuracy of 0.9691, sensitivity of 0.7948, specificity of 0.9860, AUC of 0.9845, and F1 score of 0.8172. With the addition of AGRB, accuracy increased to 0.9716, sensitivity improved to 0.8151, and F1 score rose to 0.8297. The combination of FN-Hub and AGRB further improved performance, achieving an accuracy of 0.9730, sensitivity of 0.8561, and F1 score of 0.8402. When combining PFAM with AGRB, sensitivity reached 0.8584, the highest among module combinations, with an accuracy of 0.9725 and F1 score of 0.8380. The complete EFDG-UNet model achieved an accuracy of 0.9736, sensitivity of 0.8438, specificity of 0.9856, AUC of 0.9886, and F1 score of 0.8412, demonstrating the best overall performance. While the complete model’s sensitivity (0.8438) is slightly lower than the PFAM + AGRB combination (0.8584), it achieves the highest F1 score (0.8412) and maintains competitive AUC (0.9886), indicating optimal overall balance across all performance metrics.

As shown in Table 7, the ablation experiments on the STARE dataset demonstrate consistent improvements from each module integration.

**Table 7:** Ablation experiment for EFDG-UNet on Stare dataset.

Argument	Acc	Se	Sp	Auc	F1
Unet	0.9738	0.7814	0.9897	0.9892	0.8138
AGRB	0.9632	0.8505	0.977	0.9833	0.8292
PFAM + AGRB	0.9633	0.8661	0.9752	0.9840	0.8321
FN_Hub + AGRB	0.9638	0.8534	0.9773	0.9837	0.832
EFDG-UNet	0.9637	0.8575	0.9769	0.9842	0.8376

The baseline U-Net model achieved an accuracy of 0.9738, sensitivity of 0.7814, specificity of 0.9897, AUC of 0.9892, and F1 score of 0.8138. After introducing AGRB, sensitivity significantly improved to 0.8505, though accuracy decreased to 0.9632. The F1 score increased to 0.8292. The combination of PFAM and AGRB further improved sensitivity to 0.8661, with F1 score reaching 0.8321. The FN-Hub and AGRB combination achieved an accuracy of 0.9638, sensitivity of 0.8534, and F1 score of 0.8320. The complete EFDG-UNet model achieved an accuracy of 0.9637, sensitivity of 0.8575, specificity of 0.9769, AUC of 0.9842, and F1 score of 0.8376, representing the best balanced performance across all metrics. On the STARE dataset, EFDG-UNet demonstrates a different performance characteristic compared to the baseline U-Net, with decreases in accuracy (0.9637 vs. 0.9738), specificity (0.9769 vs. 0.9897), and AUC (0.9842 vs. 0.9892). However, these changes are accompanied by a substantial improvement in sensitivity (0.8575 vs. 0.7814, a 9.7% increase) and F1 score (0.8376 vs. 0.8138, a 2.9% increase). This performance shift reflects the model’s optimization towards better detection of positive samples (vessel pixels), which is particularly valuable in clinical diagnosis where missing true vessels can have more serious consequences than false alarms. The improved F1 score indicates that EFDG-UNet achieves more clinically relevant performance by better balancing precision and recall on this dataset.

Across all three datasets, the ablation studies show that each module contributes to the overall performance of EFDG-UNet. The AGRB module consistently improved F1 scores and specificity, the PFAM module enhanced sensitivity particularly on DRIVE and STARE datasets, and the FN-Hub module effectively balanced sensitivity and specificity. The complete EFDG-UNet model, integrating all three modules, achieved superior overall performance on all datasets, demonstrating the synergistic effect of the proposed architecture. While dataset-specific performance trade-offs are observed (e.g., sensitivity decrease on CHASE_DB1, accuracy decrease on STARE), these are accompanied by improvements in comprehensive metrics such as F1 score and AUC, indicating that EFDG-UNet optimizes for clinically meaningful overall performance rather than individual metrics. The sensitivity decrease on CHASE_DB1 is attributed to the dataset’s unique characteristics—higher vessel thickness variability and more complex vascular structures—which make the AGRB module’s specificity-enhancing mechanism more conservative in detecting ambiguous vessel pixels, while still achieving superior F1 score (0.8469 vs. 0.8218) and AUC (0.9932 vs. 0.9898). On DRIVE and STARE datasets, sensitivity improvements of 6.2% and 9.7% respectively demonstrate the model’s robust capability in detecting vessel structures across different data distributions.

### Cross-dataset generalization

To evaluate the generalization capability of EFDG-UNet, cross-dataset experiments were conducted by training the model on one dataset and testing it on another. The results are presented in Table 8, comparing EFDG-UNet with several state-of-the-art methods including CS-Net, AACA-MLA-D-UNet, TP-Net, and HiDiffSeg.

**Table 8:** Cross-dataset generalization performance comparison.

Training set	Testing set	Method	Acc	Sp	Se	AUC
DRIVE	STARE	CS-Net ^43^	0.9629	0.9218	0.7884	0.9526
		AACA-MLA-D-UNet^44^	0.9497	0.9846	0.7098	0.9731
		TP-Net^45^	0.9573	0.9742	0.8079	0.9725
		HiDiffSeg^46^	0.9649	0.9869	**0.8244**	0.9782
		Ours	**0.9822**	**0.9909**	0.7722	**0.9822**
STARE	DRIVE	CS-Net^43^	0.9070	0.9494	0.796	0.9507
		AACA-MLA-D-UNet^44^	0.9559	0.9732	0.8079	0.9735
		TP-Net^45^	0.9582	0.9786	0.8190	0.9718
		HiDiffSeg^46^	0.9630	0.9706	0.8173	0.9610
		Ours	**0.9677**	**0.9812**	**0.829**	**0.9851**

As shown in Table 8, when trained on DRIVE and tested on STARE, EFDG-UNet achieved an accuracy of 0.9822, specificity of 0.9909, sensitivity of 0.7722, and AUC of 0.9822. Compared to the other methods, EFDG-UNet achieved the highest accuracy, specificity, and AUC. The accuracy of 0.9822 outperformed HiDiffSeg with an accuracy of 0.9649 and CS-Net with an accuracy of 0.9629. The specificity of 0.9909 was notably higher than HiDiffSeg with a specificity of 0.9869 and AACA-MLA-D-UNet with a specificity of 0.9846. However, the sensitivity of 0.7722 was lower than HiDiffSeg with a sensitivity of 0.8244 and TP-Net with a sensitivity of 0.8079.

When trained on STARE and tested on DRIVE, EFDG-UNet achieved an accuracy of 0.9677, specificity of 0.9812, sensitivity of 0.8290, and AUC of 0.9851. In this configuration, EFDG-UNet achieved the higher performance across all metrics compared to the other methods. The accuracy of 0.9677 outperformed HiDiffSeg with an accuracy of 0.9630 and TP-Net with an accuracy of 0.9582. The sensitivity of 0.8290 surpassed TP-Net with a sensitivity of 0.8190 and HiDiffSeg with a sensitivity of 0.8173. The AUC of 0.9851 was significantly higher than AACA-MLA-D-UNet with an AUC of 0.9735 and TP-Net with an AUC of 0.9718.

The cross-dataset experiments show that EFDG-UNet maintains reasonable performance when tested on different datasets. When trained on STARE and tested on DRIVE, EFDG-UNet consistently outperformed all comparison methods across all metrics. When trained on DRIVE and tested on STARE, EFDG-UNet achieved superior performance in accuracy, specificity, and AUC, though with slightly lower sensitivity. These results indicate that EFDG-UNet can effectively adapt to different data distributions and maintain robust performance, making it a reliable model for retinal vessel segmentation in practical clinical applications where training and testing data may come from different sources.

## Conclusion

EFDG-UNet achieves competitive results in retinal vessel segmentation across multiple metrics. The model balances accuracy, sensitivity, and specificity, with improved performance in capturing small vessels and segmenting background regions. Compared to baseline methods, EFDG-UNet shows improvements across multiple evaluation metrics, particularly in accuracy, AUC, and specificity.

However, EFDG-UNet also has some limitations. Although the model performs well across several metrics, its sensitivity remains lower than that of some advanced segmentation models in certain cases. This suggests that EFDG-UNet may require further optimization to enhance its ability to capture fine details, particularly for certain types of small vessels or noisy data. Additionally, despite the integration of various modules to enhance feature extraction, EFDG-UNet may still need improvements in its noise robustness when handling highly complex or noisy data.

Future research will focus on further optimizing the EFDG-UNet architecture, particularly in terms of sensitivity and noise resistance. We plan to incorporate more attention mechanisms and self-supervised learning strategies to improve detection of minute vessels and detailed regions. Furthermore, we will explore cross-dataset training and evaluation methods to assess the model’s generalization capabilities. This approach will help evaluate the adaptability of EFDG-UNet in different scenarios and prevent the model from overfitting to specific datasets.

## Data Availability

The datasets utilized in this study can be accessed via the following links: https://researchinnovation.kingston.ac.uk/en/datasets/chasedb1-retinal-vessel-reference-dataset-4/ (CHASE_DB1), https://www.kaggle.com/datasets/andrewmvd/drive-digital-retinal-images-for-vessel-extraction (DRIVE), and http://cecas.clemson.edu/~ahoover/stare/ (STARE).
